# Supplementary material for: Are carbon emissions trading and green financial instruments synergistic? -Comprehensive quantitative research based on content analysis
Source: PLoS One. 2024 Mar 7;19(3):e0298601. doi: 10.1371/journal.pone.0298601 (PMC10919601; doi:10.1371/journal.pone.0298601)
Supplement: S1 Data — (ZIP) [file pone.0298601.s001.zip › Data/地级市Z_F工作报告.zip!/║╙─╧/╞╜╢Ñ╔╜╩╨/└┤╫╘╣1⁄2╓┌║┼í╛┬φ┐╦╩2╛▌═°í┐_2013.docx]

各位代表：

　　现在，我代表市人民政府，向大会报告工作，请予审议，并请各位政协委员提出意见。

一、2012年工作回顾

2012年，面对严峻复杂形势和经济下行压力，在省委、省政府和市委的正确领导下，全市上下全力以赴稳增长、转方式、惠民生，经济社会保持了平稳发展态势。初步核算，全市生产总值完成1502亿元，比上年增长6．9％；地方公共财政预算收入107．5亿元、支出209．8亿元，分别增长12．8％和19．8％；全社会固定资产投资1035亿元，增长22．7％；社会消费品零售总额474．6亿元，增长15．5％；城镇居民人均可支配收入20610元、农民人均纯收入7518元，扣除价格因素，分别增长10％和11．7％。

（一）综合施策、强力落实，促进了经济平稳增长。加强经济形势分析研判，从关键环节和紧迫任务入手，迅速行动，及时研究出台了稳增长的29条措施，全力化解经济下行压力。以扩大投资拉动增长。开展项目集中开工和建设攻坚活动，309个重点项目完成投资456亿元，占年计划的168％，建成项目73个、新开工项目103个；争取上级资金11亿元，强化了投资支撑。以激活民营经济助推增长。落实结构性减税等扶持小微企业政策，新增市场主体1．2万个、新入库规模以上工业企业63家，非公经济增长10％，比重提升1．6个百分点。以优化环境服务增长。开展了为期5个月的企业环境集中整治专项行动，查处涉企案件900多起。全力做好要素保障，新增贷款140亿元，直接融资110亿元，报批各类建设用地3．5万亩。以帮扶企业稳定增长。强化产销对接、银企对接，短时间内相继召开多场企业座谈会，对反映的问题认真协调督办解决。平煤神马集团、舞钢公司、鲁阳电厂等骨干企业产销回升，经济企稳迹象更加明显。

（二）构建载体、优化结构，推动了发展方式转变。从调优存量、扩大增量两方面入手，加快产业转型升级。产业发展载体更加完善。以10个省级产业集聚区为载体，实施提升行动计划，完成投资448亿元，增长17．2％；矿山设备制造、医疗器械、新能源新材料等产业集群发展态势明显。以11个商务中心区和特色商业区为载体，坚持规划编制报批和招商引资同步推进，谋划开工一批商业地产和城市综合体项目，促进现代服务业集聚发展。以高标准粮田“百千万”建设工程和农业产业化集群培育工程为载体，大力发展现代农业。建设高标准粮田19万亩，粮食总产203万吨，连续九年丰收。新增市级以上龙头企业54家、农民专业合作社514家，中药材、面品、乳品和饮品三个产业化集群销售收入分别超40亿元、20亿元和10亿元。结构调整步伐更加坚实。建成亿元以上工业转型升级项目22个，装备制造、新型建材产业比重分别提升1．4个和0．6个百分点，六大高耗能行业比重下降1．3个百分点。文化旅游业快速发展，接待游客人数、旅游总收入增速均在30％以上；金融业稳健发展，农信社达标升级全面完成，中信银行、平顶山银行洛阳分行开业，新引进保险公司3家；大香山现代物流配送中心等服务业项目建成，三产比重提升2．8个百分点。以高效经济作物、现代畜牧业和林果业为重点，调整农业结构，烟叶生产实现历史性突破；新建标准化养殖小区236个，重大动物疫病防控、畜产品质量安全和畜牧兽医综合执法体系建设走在全省前列。科技支撑作用更加突出。15项重大技术取得突破，获国家科技进步奖3项。专利申请授权1135件，增长29％。新增国家高新技术企业6家、省级工程技术研究中心7家。质量兴市战略深入推进，鲁阳电厂获河南首个国家优质工程金奖，“圣光”品牌荣获中国驰名商标，新增省名牌产品6个，3家企业获市长质量奖。节能减排成效更加明显。深化大气环境和白龟湖综合整治，大力淘汰落后产能，主动牺牲一些眼前利益和发展速度，换来生态环境改善和长远可持续发展。全市万元生产总值能耗下降7．9％，市区空气质量优良天数327天，城市集中饮用水源地水质达标率100％。完成生态造林13．6万亩。

（三）强化引领、完善功能，提升了城乡建设水平。全市城镇化率44．9％，提高1．8个百分点。规划体系不断完善。新区总体规划正在编制，城市组团式发展规划和沿湖城市设计方案已经完成，中心城区控制性详细规划实现全覆盖，新型城镇体系初步构建。城乡建设步伐加快。15亿元城投债券成功发行。建设路提档升级、湖滨景观带亮化、高速出入口绿化、供水供热管网建设和一批“创卫”、“创森”工程完工，更新公交车100台，天然气置换人工煤气4万户。城中村和旧城连片开发改造新开工260万平方米、主体完工200万平方米。市博物馆、城市规划展示馆按期向市民开放。县城基础设施和规模不断提升。出台了加快中心镇建设的意见。新启动新型农村社区101个，累计启动313个，建设新民居3．4万套，入住1．1万户。城市管理力度加大。市数字化城管系统建成并投入运行。数字地理空间框架建设及应用示范项目通过验收，获“全国数字城市建设示范市”。持续“创卫”攻坚，开展“五个一”竞赛活动，加强城乡环境综合整治，环境面貌明显改观。

（四）深化改革、扩大开放，增强了发展动力活力。以改革创新破解瓶颈制约，以承接产业转移借力发展。体制机制创新深入推进。完成国企改制2家、正在推进的有6家，市直机关所属企业脱钩改制基本到位，6家企业入围省重点上市后备企业。事业单位改革有序展开。文艺院团改革完成。医药卫生体制改革深入推进。集体林权制度改革取得阶段性成果。新增土地流转面积11万亩。被确定为全国工矿废弃地复垦利用试点市。市公共资源交易中心启动运行。开放招商富有成效。成功举办第四届“华合论坛”，我市签约项目48个、金额530亿元，规模和成效均超往届。全年实际到位市外资金645亿元，增长23％；实际利用外商直接投资3．7亿美元，增长21．4％。

（五）以人为本、民生优先，保持了社会和谐稳定。“十大民生工程”全部完成，财政用于民生的支出达73％。安全生产形势持续稳定。把安全生产作为最大的民生，实行行政首长负责制，建立健全10大安全生产制度和34个部门安全生产责任制，深入开展煤矿、非煤矿山、消防等重点行业和领域专项整治，强力推进煤炭企业兼并重组，省、市、县及企业投入15．6亿元，关闭劝退矿井103座，地方煤矿由整合前的160座减少到57座，实现了由乱到治的根本转变，各类事故起数和死亡人数大幅下降，连续两年被评为全省安全生产优秀市。“三就两保”实现“扩面提标”。城镇新增就业10．3万人，农村劳动力新转移就业9．1万人。进一步提高了城镇居民医保、新农合筹资标准和补助水平，城乡居民养老保险制度实现全覆盖。为企业退休人员人均月增养老金221元，为7．9万城市居民和18．1万农村居民发放低保金4．3亿元。提高了公务员津补贴和事业单位绩效工资水平。建立了高龄老人补贴制度。建成保障性住房1．5万套，超额完成省定任务。农村生产生活条件不断改善。北汝河治理郏县城区段和24座小型水库除险加固工程竣工。解决农村饮水安全人口25．3万人，新建改建农村公路300公里，新增农村沼气工程15座，改造农村危房6500户，6．1万贫困人口脱贫。移民迁安工作受省表彰。社会事业全面进步。新建、改扩建幼儿园89所、市区小学18所。全国职教技能大赛获金牌全省第一。两所本科院校通过国家教学评估。年度文化惠民工程全面完成。争取城乡医疗卫生服务项目186个，建成了一批乡（镇）卫生院、急救指挥中心和卫生监督所。全民健身运动广泛开展，竞技体育实现了奥运金牌零的突破。人口计生工作继续保持全国先进。外事、宗教等工作获全国先进称号。社会大局和谐稳定。健全群众工作体系，十八大期间信访稳定工作受省表彰。中心城区104个社区97个达到和谐社区标准。深入推进平安建设，社会管理综合治理得到强化，人民群众安全感进一步提升。

同时，加强民主法制建设，自觉接受人大监督、政协民主监督和社会监督，认真办理人大代表建议和政协委员提案。坚持依法行政，加强行政监察，深入实施“六五”法制宣传教育和依法治市工作。加强精神文明建设，涌现出了马俊欣、李博亚等一批先进典型。大力开展国防教育，驻平解放军和武警部队在支持地方建设中做出重要贡献，拥军优属、拥政爱民取得新成效。

总的看，一年来，我们在逆境中攻坚克难，拼搏奋进，集中精力解决了一批影响当前发展的急事，集中财力办好了一批为民惠民的实事，集中力量干成了一批事关城市竞争力的大事，集中优势推进了一批打基础利长远的要事。这一年，我们最强烈的共识，就是推动科学发展必须加快调结构、转方式；最深切的感受，就是抓工作必须深入具体、盯住不放；最欣慰的成果，就是在困难情况下保障改善了民生，城乡居民收入保持两位数增长；最难得的突破，就是全市公共财政预算收入和支出分别突破100亿元和200亿元，全社会固定资产投资突破1000亿元，经济社会发展站上了新的起点。

各位代表！

回首过去的一年，充满了艰辛和不易，饱含着全市人民的辛劳和汗水。在此，我代表市人民政府，向辛勤工作在全市各条战线的广大干部群众，向所有关心、支持、参与平顶山建设发展的海内外朋友，表示衷心感谢并致以崇高的敬意！

在总结工作的同时，我们也清醒认识到，我市发展速度不快、产业结构不优、投资规模偏低、市场主体偏少、集聚区承载能力不足、体制机制障碍等问题仍较突出，经济抵御市场风险的能力较弱，支柱产业、骨干企业尚未走出困境，个别指标未能实现年初预期，经济发展面临的形势依然严峻；公共服务和城乡基础设施欠账较多，一些群众生活仍较困难，改善民生需要付出更艰辛的努力；领导方式和政府职能转变还不到位，部分干部责任意识不强、履职能力不足、工作作风不实，回避矛盾，不作为、乱作为，铺张浪费、消极腐败现象还不同程度存在。我们要正视问题，并认真剖析问题背后的原因，采取切实有效措施加以解决。

二、2013年工作目标与任务

各位代表！

我们深知，新的一年，人民群众对政府工作充满着新的期盼。期盼我们保持经济持续增长，提供更加充分的就业创业机会，让收入更高、财富更多；期盼我们完善城市功能，让出行更顺畅，生活更便利；期盼我们健全公共服务体系，提供更多更好的教育、文化、医疗、住房保障等服务，让困难群众得到更多的关怀和帮扶；期盼我们下大功夫解决大气环境、水环境等突出问题，让鹰城的天更蓝、水更净、空气更清新、城市更宜居。这些期盼，既是广大人民群众对美好生活的向往，也是政府工作的目标和重点，我们将为之不懈努力！

今年政府工作要深入贯彻落实党的十八大和市委经济工作会议精神，坚持以邓小平理论、“三个代表”重要思想、科学发展观为指导，紧紧围绕主题主线，以提高经济增长质量和效益为中心，立足新起点、构建新平台、争创新优势，坚持学先进、比创新、看实效，正气实干，转中求进，在加快信息化进程中更加积极地探索“两不三新”三化协调科学发展之路，努力实现经济社会持续健康发展。重点是：围绕“一个总目标”，实现“一个新突破”，打好“一场攻坚战”，展示“一种新形象”。“一个总目标”就是提前全面小康、建设美丽鹰城。去年，我市全面小康23项指标综合实现程度83．5％，高于全省平均水平；差距较大的有6项。要把各项指标科学分解落实到年度、部门、责任人，制定居民收入、三产比重、单位GDP能耗、研发经费投入比重等薄弱指标提升行动计划，作为考量工作成效的重要标尺。“一个新突破”就是新区规划建设要有新突破。建设新区是平顶山实现可持续发展的重要载体和历史机遇，必须举全市之力，按照“三区一中心”的功能定位和详细规划，严格控制，积极推进，尽快完善基础设施条件，促其出形象、上规模。“一场攻坚战”就是打好产业转型升级攻坚战。围绕转型多谋划，围绕转型抓招商，围绕转型上项目，使今年成为项目大开工、投资大增加、产业大提升的重要一年。“一种新形象”就是以干部作风转变展示为民务实清廉的形象，让人民群众切身感受到政府在用心干事、勇于担当。

经济社会发展主要目标是：生产总值增长10％左右；地方公共财政预算收入增长10％以上；全社会固定资产投资增长22％；社会消费品零售总额增长16％；外贸出口增长15％；实际利用外商直接投资增长10％；城镇居民人均可支配收入、农民人均纯收入分别实际增长9％以上，力争与经济增长同步；城镇新增就业7．5万人；人口自然增长率控制在6．5‰以内；居民消费价格涨幅控制在3．5％左右；城镇化率提高1．8个百分点；节能减排完成省下达任务。

围绕上述要求和目标，着力做好八个方面工作：

（一）加快产业转型。我市作为典型的资源型城市，要实现可持续发展，调整结构、转型升级势在必行。今年要以实施100个亿元以上传统产业升级和100个亿元以上新兴产业培育项目为抓手，深化企业服务，争创产业新优势。调整优化存量。对煤炭、电力、化工、钢铁等传统优势产业，加大帮扶力度，搭建好产销对接、银企对接服务平台，帮助企业开拓市场，增加订单，释放产能。利用市场倒逼机制，加快产业产品结构调整，重点抓好平煤神马25万吨己二酸和20万吨己内酰胺、海星化工10万吨针状焦、厚普盐化120万吨真空制盐、中加矿业300万吨铁精粉等项目建设，不断向下游精深加工延伸，巩固扩大传统产业优势。着力扩大增量。装备制造产业，重点抓好智能电网高端装备产业基地、煤炭综采成套装备及智能控制系统、隆鑫机车工业园等项目，装备制造板块主营业务收入超400亿元。围绕新兴产业，重点抓好圣光医疗孵化园、兴宇中科锂电池隔膜等项目，形成一批超100亿元的优势产业集群和超20亿元的特色产业集群。提升产业集聚区水平。每个产业集聚区都要围绕各自主导产业，引进国内外知名企业，形成龙头带动效应。集聚区全年完成固定资产投资550亿元，其中，基础设施和综合配套设施投资100亿元；规模以上工业主营业务收入超1000亿元。完善集聚区升级晋位考核办法，推动高新区创建国家级高新区。增强创新驱动发展能力。加大科技投入，实施重大科技专项，新增省级工程技术研究中心5家，争取在国家重点实验室建设上取得突破。新增国家高新技术企业6家、省以上名牌产品10个，制定国家标准5项。专利申请1600件以上，争创国家知识产权示范市。支持高新区国家级科技企业孵化器建设。加快移动无线城市、3G网络扩容扩建和“智慧平顶山”建设，促进信息化与工业化深度融合。加快发展现代服务业。全面完成商务中心区、特色商业区空间规划和控制性详规编制，实施“两区”建设项目23个，带动商务、信息、创意等现代服务业发展。积极引进金融主体，支持农信社组建农村商业银行，壮大金融产业规模。强力推进旅游目的地建设，精心打造尧山—大佛景区、大香山风景名胜区等一批旅游景区和精品线路，加快游客服务中心建设，培育3－4家大型旅游企业，叫响“中原大佛、神奇尧山、观音祖庭、近悦远来”旅游品牌。积极推进平东物流中心、国药配送中心等项目建设，健全流通网络，激活各类消费，提升服务业比重。

（二）主攻项目建设。安排重点项目300个，总投资2600亿元，年度投资500亿元。其中，计划建成平高大容量强电流试验站、平煤神马2万吨高性能浸胶帘子布等83个项目；续建宝隆不锈钢产业园等91个项目；新开工中材环保10万吨袋式除尘器等108个项目；积极推进叶县盐腔储气库等18个前期项目。对项目建设实行联审联批、跟踪督查、通报奖惩等制度，下大力气解决土地、资金、环境容量等突出问题，以项目扩投资、强支撑。

注重加强基础产业和基础设施建设，为长远发展备足后劲。能源方面，完成平煤神马朝川矿升级改造，建成平姚地下输煤通道；加快平煤神马夏店矿、龙口220KV输变电等工程进度；开工一批风电场等新能源项目；推进姚电3、4号机组热电联产技改，优化能源结构，提升保障水平。交通方面，重点抓好平郏、宝石、神马大道东延等快速通道建设和国道311改建，支持郏县地方铁路、平西火车站广场改造、孟平铁路复线电气化改造、焦桐高速汝州至登封段等项目建设，积极推进郑万客运专线、许平洛城际轨道交通、尧山机场、大南环等重大项目前期，提升区位交通优势，打造豫中南综合交通枢纽和物流中心。水利方面，完成孤石滩水库和21座小型水库除险加固以及北汝河综合治理四期、鲁山大浪河、湛河区大泥河治理等工程；加快南水北调受水区城市供配水工程建设，提高水资源保障和防洪抗旱能力。

（三）统筹城乡发展。全域推进中心城市、县城、中心镇、新型农村社区四级城镇体系建设，拉动内需、承载发展。全面加快新区建设。成立新区建设领导小组，组建新区管委会。上半年完成新区总体规划评审，加快编制控制性详规。强力推进主干路网、新区人民医院、商场、学校等基础设施建设，完成引昭入市工程设计并启动实施，开工建设投资100亿元的建业城市综合体、投资120亿元的天明国际城、投资240亿元的御景半岛休闲商务中心，争取开工投资35亿元的经贸中心，加快和盛广场建设进度，筹建人大政协综合楼。全年完成投资175亿元，完善功能，提升品质，集聚商气人气。推进老城区提档升级。对重点区域和重要节点开展城市设计，完成供热、燃气等专项规划，启动地下空间的规划控制和综合利用工作。着力“建环线、打卡口、接断头”，实施道路工程11项，建成凌云路南延、一矿路改建、光明路北延等工程，推进平安大道西延、湛南路湛北路西延、开发一路北延等工程，提高道路通行能力。完善市政设施，延伸供水、排污、燃气、供暖等管网，实现东部、北部全覆盖。继续推进湛河上游治理，启动市内重点水系建设，活化水体，改善水质。加快湛南新城建设，完善配套设施，尽快形成建设高潮。继续抓好城中村和旧城连片开发改造，开工260万平方米；推进鹰城国际商务中心和新华园怡购城等城市综合体建设。充分发挥数字化城管平台作用，实现城区数字城管全覆盖。建设改造8个市内农贸市场。新增城市绿地158万平方米。合力攻坚，确保今年“创森”成功、“创卫”通过国家暗访，并形成城市管理长效机制。增强县城和中心镇承接承载能力。以快速通道建设为突破口，推动中心城区与鲁山、叶县、石龙组团式发展，引导产业分层布局、功能互补。推进汝州、舞钢、郏县三个卫星城向区域性中心城市发展。支持各县（市）抓好新区开发、老城改造、产业集聚区、商务中心区和特色商业区建设，构建发展载体，提升县域经济综合实力。每个县（市）重点抓好1至2个示范镇，明确建设标准，突出产业特色，注重典型示范。促进人口向城镇转移。坚持产业为基、就业为本、住房牵动和学校牵动，解决好农民进城就业、户籍、社会保障、子女入学等问题，有序推进农业转移人口市民化。抓好新型农村社区建设。持续在新社区建设中的有益探索，按更高标准再启动一批新社区。已启动的要扩大规模、完善设施、搞好服务、引导入住。探索开展人地挂钩试点，争取成为省级试点市；建立土地指标交易平台，破解建设资金瓶颈。加快旧址拆迁复耕，争取有大的突破。

（四）发展现代农业。坚持保粮与增效并重，全面落实强农惠农富农政策，提升农业现代化水平。推进高标准粮田“百千万”建设工程。围绕提高粮食综合生产能力，加快以抗旱除涝为重点的农田水利工程建设，统筹推进道路、电力等生产服务设施建设，建成高标准粮田20万亩，粮食总产稳定在200万吨以上。抓好现代农业产业化集群培育工程。鼓励和支持承包土地向专业大户、家庭农场、农民专业合作社流转，加快建设无公害、绿色和有机农产品生产基地，推进主食产业化，集中力量培育面品、肉品、乳品和饮品、蔬菜、中药材、调味品和柞蚕等7大类共10个具有地方特色的农业产业化集群。新增土地流转面积10万亩、农民专业合作社300家，新建标准化养殖小区200个，培育循环畜牧企业50家，市级以上龙头企业达到200家。加快建设现代农业服务体系。建成市农产品检验检测中心、气象科技园等项目，扩大农技推广、良种培育、动物防疫和农产品质量检测覆盖范围，加快实现主要农作物生产全程机械化。扶持培养职业化新型农民。不断改善农村生产生活条件。解决20万人饮水安全问题，新改建农村公路200公里，完成新一轮农村电网改造升级。创新扶贫开发机制，实现5万人口脱贫。做好各类实用技术培训和劳动力转移培训，新增转移就业5万人。

（五）深化改革开放。把对外开放作为应对复杂局面和破解各种制约的综合性战略举措，以开放促改革、促发展。大力承接产业转移。整合招商资源，理顺招商体制，完善激励机制，以产业链招商为主，开展专业化集群式招商，注重引进龙头基地型项目，全年实际到位市外资金690亿元以上。盯紧“华合论坛”等已签约项目落地，创造条件让招商项目早日开工建设。积极拓展开放领域。加快服务业、社会事业领域开放步伐，引进社会资本办医院、办学校、办养老机构。落实支持民营经济发展的各项政策措施，实施中小微企业成长工程，鼓励引导从煤炭行业退出的民间资本投入转型领域。积极扩大出口。扎实推进各项改革。鼓励支持企业跨行业、跨地域、跨所有制战略合作，推进平煤机与三一重工重组，年内基本完成星峰集团破产改制，加快国有商贸企业改制步伐。深化财税体制改革，研究“营改增”政策对我市的影响，加强市级财源建设。深化事业单位分类改革。完成县级公立医院改革评估。继续精简审批事项，提高行政效能。创新要素保障机制。强化节约集约用地，扎实开展土地综合整治；组建土地收储中心，增加土地储备，破解用地制约。加强银企合作，做大投融资平台，支持平煤神马财务公司组建运营，支持巨龙、圣光等企业上市，力争社会融资规模达到240亿元。启动城投债券二期工作。加强信用担保机构规范整顿，防范化解金融风险。

（六）推进文化强市。用文化滋养城市，提升文化软实力。大力繁荣文化事业。优化文化资源配置，创新管理运行方式，提高公共文化服务水平。继续实施舞台艺术送农民、农村（校园）公益电影放映、农家书屋、广播电视“村村通”等文化惠民工程。完成市民活动中心选址立项、高山无线发射站建设和数字电视整体转换，开工建设市老干部活动中心。做好县级“两馆”达标工作。大力发展文博事业，推进应国墓地及宝丰清凉寺汝官窑遗址公园建设。加强传统村落保护。加快发展文化产业。设立文化产业发展基金，推进宝丰文化改革发展试验区建设，加快文化创意产业园、汝瓷文化园、大香山国学文化园等项目建设，着力培育一批文化产业园区和文化品牌。围绕陶瓷、金镶玉、绢花、奇石、根雕等传统工艺，开发文化旅游商品。健全文化管理体制和文化生产经营机制，激发报纸期刊、演艺娱乐、广播电视等行业活力。提高市民文明素质。深入开展社会主义核心价值体系学习教育，着力推进公民道德建设工程，广泛开展志愿服务，培育良好风尚。持续“守规则、尊重人、争当文明市民”和道德模范评选等活动，深化群众性精神文明创建，争创省级文明城市。

（七）建设生态文明。牢固树立绿色循环低碳发展理念，努力建设美丽鹰城。强化资源能源节约。实行能源消费总量预算管理，建立碳排放控制制度，推行节能量交易。继续深化工业、交通、建筑和公共机构节能，开展合同能源管理，突出抓好重点行业、重点企业节能技术改造。坚决淘汰落后产能，对已列入淘汰计划的企业和生产线，限期关停。以煤层气、焦炉煤气、矿井水、煤矸石、粉煤灰综合利用为重点，大力发展循环经济，加快培育一批循环经济示范企业和示范园区。加强再生资源回收利用一体化经营体系建设。加大环保整治力度。实施燃煤机组除尘脱硝改造，取缔城区35吨以下锅炉。加强扬尘、粉尘、机动车尾气、餐饮油烟等污染防治，认真开展PM2．5测报工作，市区空气质量优良天数保持在300天以上。推进污水、污泥处理和生活垃圾填埋等工程。加大对重点水污染源的治理和监管力度，确保出境水质达标。加强白龟湖区域及上游四条河流的环境综合整治，对入湖河流实行生态补偿机制，确保城市集中饮用水源地水质达标率100％。大力开展农村环境连片整治，努力解决“垃圾围村”问题。加快林业生态建设。实施山区生态体系、农田防护林、生态廊道等8项重点林业工程，新造林地8．9万亩。编制北部矿区综合整治总体规划，推进矿区生态修复和煤矿沉陷区综合治理。启动白龟湖国家湿地公园建设，规划建设沙河生态景观带。

（八）保障改善民生。继续办好“十项重点民生工程”，让群众得到更多实惠。突出抓好安全生产。做好煤矿兼并重组后续工作，妥善处理遗留问题。严格落实行政首长负责制，强化煤矿、非煤矿山、道路交通、消防、建筑施工、食品药品、危险化学品、烟花爆竹、商场、学校等重点领域和人员密集场所的安全监管，确保安全生产形势持续稳定。推动实现高质量稳定就业。实施全民技能振兴工程和公益性岗位安置计划，重点做好高校毕业生、城镇困难人员、退役军人就业，动态消除“零就业家庭”。落实国家收入分配制度改革政策，健全职工工资正常增长和支付保障机制。全面提升社会保障水平。积极推动城镇职工养老保险与城乡居民养老保险衔接，城镇职工医保与居民医保、新农合衔接。提高城镇居民医保、新农合政府补助标准和实际报销水平。保障企业退休人员基本养老金调整到位，提高城乡低保、农村五保供养标准和优抚对象补助水平。实施敬老院“温暖工程”，开工建设未成年人保护中心、儿童福利院和夕阳红老年公寓扩建项目。加强保障性安居工程建设与管理，重点发展公共租赁房，新开工保障性住房1．45万套。促进老龄、红十字、残疾人和慈善事业发展。大力发展社会事业。落实学前教育三年行动计划，新建、改扩建幼儿园115所。开工市四十一中移址重建项目。启动市委党校新址建设。支持市高级技工学校建成国家级重点技师学院，推进职业教育园区规划建设。以教学评估为契机，提升高等教育水平。完善医疗卫生服务体系，建成10个乡镇卫生院、4个县级急救指挥中心和8个县级卫生监督机构，加快市精神卫生中心、儿童医院等项目进度，推进健康城市建设。完善人口计生政策服务体系，稳定低生育水平，提高出生人口素质。广泛开展全民健身运动，完成市体育村主要场馆升级改造。加快公共停车场、公交场站建设，更新公交车辆，方便群众出行。创新社会管理。健全维护群众权益机制，做好信访工作。中心城区实现和谐社区全覆盖，新创建“四无”乡镇（街道）20个。创新流动人口、特殊群体、网络虚拟社会的管理和服务。加强应急管理，提高突发事件应对处置能力。深化平安鹰城、法治鹰城建设，强化社会管理综合治理，保持社会大局和谐稳定。

加强国防教育和国防后备力量建设，支持驻平解放军、武警部队、预备役部队、民兵和人民防空建设，深入开展双拥共建，促进军民融合式发展。支持工会、共青团、妇联、科协、工商联等人民团体发挥桥梁纽带作用，注重发挥社团作用。搞好第三次经济普查。继续做好外事、侨务、对台、民族、宗教、史志、盐业、地震、档案、国家安全等工作。

各位代表！

任务已经明确，关键在于落实。全体政府工作人员，各级领导干部，首先是市政府领导班子和组成人员：

必须强化责任意识。责任心是决定成败的关键，尤其在困难和挑战面前，主动负责、敢于担当是一种品格和境界。我们一定要牢记党的重托和人民的期盼，时刻不忘肩负的职责，在招商引资、项目建设、企业服务上，不管遇到什么样的问题，都要马上就办，贴心服务，以企业家的评价和知名企业落户来评判我们的发展环境；在违法建筑拆除、“双创”攻坚、环境整治、历史遗留问题化解中，不管遇到什么样的矛盾，都要勇于面对、敢于碰硬，真正对党负责、对全市人民负责、对这座城市的历史和未来负责。

必须提升履职能力。有效履行岗位职责需要能力，我们不仅要主动作为想干事，而且要有效作为能干事、干成事。要加强学习，深入研究，把握运用规律，坚持依法行政，注重战略谋划，提高科学、民主决策能力。要抓住事物主要矛盾和矛盾的主要方面，调动各方力量，创造性工作，提高科学运作、有效运作能力。要坚持关键在做，对议定的事、看准的事，立说立行、一抓到底，提高工作推动力和执行力。修订完善干部实绩考核办法，加大行政效能督查力度，以“学比看”要求，自我加压、奋力争先。

必须树立务实作风。带头落实中央改进工作作风、密切联系群众的八项规定和省委、市委贯彻意见，健全并认真落实一系列有效的重点工作、重点项目和经济运行督促检查协调制度，大力优化发展环境，向作风转变要推动工作的“正能量”。始终把人民群众放在心中最高位置，多到情况复杂、矛盾集中、发展困难、群众意见大的地方去，帮助解决问题。认真落实廉政建设责任制，加强廉政教育，完善惩防体系，加大监察、审计监督力度，坚决惩处以权谋私、权钱交易等违纪违法行为；厉行勤俭节约，反对铺张浪费，力戒虚俗假贪，展示为民务实清廉的新形象。

各位代表！

作为人民的政府，我们一定严格执行人大及其常委会的决议决定，自觉接受法律和工作监督，主动接受政协民主监督，认真办理人大代表建议和政协委员提案，广泛听取各民主党派、无党派人士、人民团体意见，虚心接受社会监督和舆论监督，让人民更有效地监督政府，使政府更好地服务人民！

各位代表！

发展重任在肩，形势催人奋进。让我们在市委的坚强领导下，坚定信心，迎难而上，务实重干，奋发有为，为提前全面小康、建设美丽鹰城而努力奋斗！
